# Applications of High-Throughput Clonogenic Survival Assays in High-LET Particle Microbeams

**DOI:** 10.3389/fonc.2015.00305

**Published:** 2016-01-25

**Authors:** Antonios Georgantzoglou, Michael J. Merchant, Jonathan C. G. Jeynes, Natalie Mayhead, Natasha Punia, Rachel E. Butler, Rajesh Jena

**Affiliations:** ^1^Department of Oncology, Addenbrooke’s Hospital, University of Cambridge, Cambridge, UK; ^2^Manchester Academic Health Science Centre, Institute of Cancer Sciences, University of Manchester, The Christie NHS Foundations Trust, Manchester, UK; ^3^Centre for Biomedical Modelling and Analysis, University of Exeter, Exeter, UK; ^4^Ion Beam Centre, University of Surrey, Guildford, UK; ^5^Department of Microbial and Cellular Sciences, University of Surrey, Guildford, UK

**Keywords:** clonogenic survival assay, high-LET radiation, microbeam, bright-field imaging, cell tracking

## Abstract

Charged particle therapy is increasingly becoming a valuable tool in cancer treatment, mainly due to the favorable interaction of particle radiation with matter. Its application is still limited due, in part, to lack of data regarding the radiosensitivity of certain cell lines to this radiation type, especially to high-linear energy transfer (LET) particles. From the earliest days of radiation biology, the clonogenic survival assay has been used to provide radiation response data. This method produces reliable data but it is not optimized for high-throughput microbeam studies with high-LET radiation where high levels of cell killing lead to a very low probability of maintaining cells’ clonogenic potential. A new method, therefore, is proposed in this paper, which could potentially allow these experiments to be conducted in a high-throughput fashion. Cells are seeded in special polypropylene dishes and bright-field illumination provides cell visualization. Digital images are obtained and cell detection is applied based on corner detection, generating individual cell targets as *x–y* points. These points in the dish are then irradiated individually by a micron field size high-LET microbeam. Post-irradiation, time-lapse imaging follows cells’ response. All irradiated cells are tracked by linking trajectories in all time-frames, based on finding their nearest position. Cell divisions are detected based on cell appearance and individual cell temporary corner density. The number of divisions anticipated is low due to the high probability of cell killing from high-LET irradiation. Survival curves are produced based on cell’s capacity to divide at least four to five times. The process is repeated for a range of doses of radiation. Validation shows the efficiency of the proposed cell detection and tracking method in finding cell divisions.

## Introduction

Charged particle therapy is increasingly becoming a valuable tool in cancer treatment, mainly due to the favorable interaction of particle radiation with matter: it maximizes the dose attributed to a specific depth of tissue by adjusting the beam energy and intensity, creating a peak of dose called Bragg peak (1). Although thousands of patients have been already treated with particle therapy during the last 60 years, uncertainties still limit the application of this treatment method. One of the limiting factors is the lack of correlation between the delivered dose of radiation and the biological output (2). Clinical trials boost the knowledge and experience in handling particle therapy situations but data are limited. However, working with high-throughput *in vitro* biological cell assays can provide valuable information regarding the interaction of single cells with charged particle radiation (3).

## Clonogenic Survival Assay

### Basic Principles

Cell radiosensitivity can be examined by performing a clonogenic survival assay *in vitro*. The clonogenic integrity post-irradiation is examined by the ability to divide and form colonies of at least 50 cells (4). The outcome is the correlation between deposited radiation dose and biological end-point investigated. The basic principles of this tool are well-manifested in the literature (4, 5); therefore, only a short overview will follow. Biological cells are seeded in a number of dishes and each dish is irradiated with a known type of radiation with different dose for every dish. One or more dishes are not irradiated (control dishes) and these are used to calculate the plating efficiency (PE). The ultimate goal of a clonogenic survival assay is the production of a graph in which the logarithmic survival fraction (SF) is correlated with the dose.

### Radiobiological Models

Although different models have been proposed to describe the relation between cell SF and dose, the linear-quadratic (LQ) model is widely recognized although questioned over its universal fit. According to this model, the cell survival curve exhibits a linear decrease with dose for lower doses while it has a steeper fall-off for higher doses (i.e., quadratic), expressing a higher impact from high-dose radiation to cells. Eq. 1 provides the formula that correlates the dose with the SF:

(1) $$SF=e^{\left(-\alpha D-\beta D^{2}\right)}$$

where α (Gy^−1^) and β (Gy^−2^) are the cell radiosensitivity parameters (6), specific for a particular experiment and cell type. The ratio α*/*β gives the dose (Gy) where both components, linear and quadratic, have equal contribution to cell survival.

Nevertheless, at low doses, data are not reliable due to low cell killing probability and survival rates are generated through extrapolation toward zero-dose (7). However, mammalian cells’ increased radiosensitivity in very low doses (<10 cGy) result in enhanced cell killing (8, 9) and, hence, an Induced-Repair term has been suggested to correct for the adverse cell response in low doses; Eq. 1 becomes Eq. 2:

(2) SF=e(−αD(1+(αsα−1)e−DDc)−βD2)

where α*_s_* is the slope of the low-dose curve of the corrected model, while *D_c_* is the dose at which cells start to become radioresistant (10). Besides low doses, the LQ model may overestimate the irradiation effect at doses >5–6 Gy (7).

Apart from the LQ model, the local-effect model has been introduced. This model is based on the notion that cell inactivation is caused almost entirely by ion traversals in the local area of cell nucleus and it depends only on the number and proximity of those traversals (11, 12). The effect is independent to radiation type with equal doses causing equal effects; therefore, the radiobiological effect from charged particle radiation can be derived from the respective effect from photon radiation (13). According to this model, the SF is described by Eq. 3:

(3) $$-\ln\text{SF}=\{\begin{array}{r} \alpha_{X}D+\beta_{X}D^{2},D\leq D_{t}\\ \alpha_{X}D+\beta_{X}D^{2}+s_{\max}(D-D_{t}),D>D_{t} \end{array}$$

(4) $$s_{\max}=\alpha_{X}+2\beta_{X}D_{t}$$

where *s_max_* is the maximum slope, α*_X_* and β*_X_* are the slopes for the photon LQ model and *D_t_* is the threshold dose above which the SF decreases exponentially (11).

### Cell Survival Studies with High-LET Radiation

Cell survival depends strongly on the linear energy transfer (LET) of the beam that is the radiation energy deposited in matter per unit of distance. Research so far has indicated that high-LET radiation (generally LET >10 keV/μm) is more effective in cell killing with the survival curve being much steeper than in low-LET radiation. Since the beginning of 1960s, it was shown that high-LET α-particles produce an exponential kidney T_1_ cell survival curve that becomes linear and steep for higher doses (14). Low-energy high-LET protons produced lower SF in V79 Chinese hamster cells (15), while high-LET α-particles produced clustered DNA damage (16). High-LET carbon ions resulted in as low as 5% survival of AG1522D cells in experiments at GSI (17) when five particles hit each cell. This evidence is strongly supported by experiments in NIRS which showed that high-LET carbon ions are more effective in killing human colon cancer stem-like cells (18), pancreatic cancer stem-like cells (19), or A549 lung cancer cells and human embryonic kidney cell than low-LET X-rays (20). Moreover, high-LET α-particles induced a lower than 10% survival of A549 lung cancer cells for a dose of 2 Gy compared to the respective rate of higher than 50% for X-ray irradiation (6, 21).

### Drawbacks of Existing Method

Although clonogenic survival assays are used widely to quantify radiation effects, there are some practical complications. First, in some laboratories, cells are seeded into special chambers that fit into the charged particle facilities. Following irradiation, cells have to be detached and re-seeded to normal dishes for follow-up (9), which may lead to additional cell death. Moreover, the standard protocol involves invasive cell staining methods for macroscopic colony counting, which ultimately leads to cell killing. The staining process is also characterized by difficulty in transfection for some cell lines while stains fade with time due to cellular physiological processes or even divisions. Colonies are counted after 5–6 cell divisions; depending on the specific cell cycle time, this process can be slow providing results even after 2 weeks. Additionally, when cells are irradiated with an average of one particle per cell, particle distribution follows the Poisson statistics: 37% of the cells receive the prescribed number of particles, 26% receive more than this dose while the rest 37% of the cells do not receive any dose (22).

## Clonogenic Survival Assay Using High-Let Microbeam Irradiation

In this paper, we present the theoretical base and the methodology for a new type of clonogenic survival assay for high-throughput cell irradiation, designed for high-LET targeted irradiation experiments, providing examples for its application. The proposed method focuses on the detection of mitotic catastrophe (cell death after unsuccessful attempt to divide) as a result of cell response to radiation; it does not assess the traditional colony formation potential but operates as a complementary technique. This method involves the precise irradiation of numerous single cells *in vitro* using a charged particle microbeam, with subsequent follow-up of cell response through label-free bright-field time-lapse imaging.

### Microbeams in Radiobiology

Although modern microbeams were originally designed for non-radiobiological experiments, they can be used to irradiate cells *in vitro*. They produce radiation beams with high spatial accuracy since their field size can be smaller than 1 μm (23, 24), enough to selectively target a cell compartment, such as the nucleus which has a typical diameter of 5–10 μm (25). They also overcome the problem of particle Poisson hit distribution of broad-beam facilities by irradiating all cells with a precise dose of a number of *N* particles, leading to uniform dose distribution.

Dosimetry in microbeam irradiation is highly important in subcellular level. The attributed dose depends on the LET, particle fluence, and cell density (9). The latter is not always stable. Although a cell is considered to have similar density to water, it is not known whether this approximation remains constant over time (26). Moreover, the change in cell thickness may well affect the delivered dose as thicker cells increase the radiation interaction and, thus, the energy deposition.

### Rationale of High-LET Clonogenic Survival

When using high-LET radiation to perform a clonogenic survival assay, the objectives are subtly distinct. High-LET radiation is densely ionizing radiation and it is responsible for complex lesions that may include several DNA bases, single-strand or double-strand breaks (25). When a molecule of DNA is traversed by a high-LET charged particle, multiple such lesions are produced (27). In many cases, the cell is unable to repair those multiple lesions while false damage identification and misrepair can also happen (28). Therefore, in high-LET irradiation, if four to five divisions occur and originate from the same cell, then there is a high probability that this cell has maintained its reproductive integrity (29). Therefore, the assessment of mitotic catastrophe can provide reliable and complementary data to colony formation assay regarding the cell response. Moreover, the investigation of clonogenic potential of the progeny could provide evidence for late-appearing effects.

### Surrey Vertical Microbeam and Secondary Microscope

The Wolfson Surrey vertical microbeam (WSVM) was used in this research as a facility that provides highly focused high-LET radiation. A complete description of this microbeam can be found in Merchant et al. (30) and Jeynes et al. (31). Therefore, only a short overview will follow. The WSVM was specifically designed for radiobiological experiments and, hence, its vertical configuration achieves minimum cell stress. It has an estimated maximum irradiation capacity of 20,000 cells per hour. The smallest achieved radiation spot size is 1 μm, which makes the beam suitable for irradiating individual cells. It provides a range of particles, from protons to calcium ions, with energies from 0.5 to 12 MeV.

On top of the beam exit, there is an integrated up-right microscopy facility that serves in cell imaging and microbeam targeting. The microscopy facility provides full environmental control to ensure optimum living conditions for the cells: temperature of 37°C, humidity of 95%, and CO_2_ flow of 5%. A three-axis motorized stage provides dish movement across all directions *x–y–z* for cell targeting. An objective water-dipping lens is mounted above the dish, while a digital camera system provides cell imaging.

However, due to difficulties in maintaining suitable environmental conditions for the cells at the microbeam microscopy facility, a secondary microscope was used to perform long time-lapse validation experiments. In those experiments, U-251 MG pleomorphic human glioblastoma (U251) cells were used. A Nikon Eclipse Ti-E confocal microscope was used in bright-field illumination mode with a Nikon CFI S Plan Fluor 40× objective.

### Principles of Suggested Method

#### Dish Preparation

The design of the cell dish that is used in most microbeams is crucial to the irradiation outcome. At the WSVM, the radiation beam has to penetrate the dish bottom in order to reach and irradiate the cells. Nevertheless, due to the low output energy, the radiation beam will strongly interact with the dish material if the latter has certain thickness. Common plastic or glass substrates with thickness in the region of 150 μm are not suitable for these experiments. Therefore, thin polypropylene foils, with thickness of 4 μm, are used as substrate material in order to avoid strong interaction between the radiation beam and the substrate (32, 33). The polypropylene foil is kept between two metallic parts and a rubber o-ring, creating a water-tight environment for the cells and the culture medium.

The seeding process was carried out as previously described (33). However, the density of cells in the dish is a factor that needs special consideration. Research has indicated that density has to be low in order to allow cells to evolve and divide, exploiting their clonogenic colony formation ability. More specifically, either very low densities of 2–8 (34), 5 (35), and 6.4 cells/mm^2^ (36) or higher densities of 120 cells/mm^2^ (37) have been accounted in the literature. Although the proposed method does not exploit cells’ clonogenic potential but rather their proliferative capacity, it was decided to follow the established protocol in cell seeding.

#### Cell Imaging

Fluorescence microscopy is the most common imaging method in microbeam community as it is used in many microbeam facilities (23, 24). However, enhanced photo-toxicity to the cells due to excess stain excitation in time-lapse imaging may lead to additional cell damage and, hence, overestimation of irradiation effect. Therefore, it has been suggested that alternative to fluorescence imaging methods should be used in clonogenic survival experiments (38, 39).

**Figure 1:**
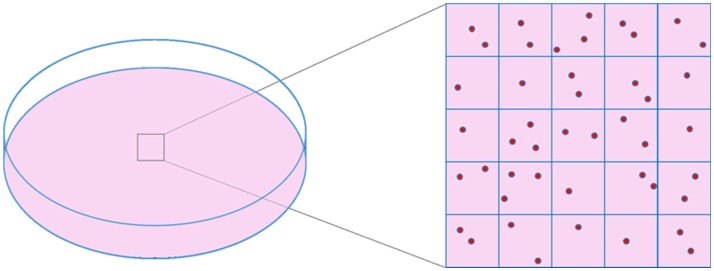
**Schematic representation of cell dish area selection and virtual division of this area into frames, based on objective’s FOV size**.

Phase contrast is an excellent alternative that offers good image quality. It has been previously used in α-particle collimated irradiation devices (40–42) or even microbeams (43) but it is difficult to implement in the WSVM vertical configuration due to conflicts with the path of the beam. Therefore, label-free bright-field illumination microscopy is used to provide cell imaging for reasons that have been justified in the literature (33, 39). Cell imaging is performed in two separate sessions. First, prior to cell irradiation, the dish is inspected under the microscope and an area containing cells is chosen. The size of this area depends on the number of cells to be irradiated. A wider area provides more targets for irradiation. The chosen area is virtually divided into field of views (FOV), depending on the FOV of the objective (Figure 1). An electrostatic scanning is then performed: the system stage-dish moves at the position of the first FOV under the objective, an image is acquired, image analysis is performed for cell target definition (i.e., *x–y* points of cell centroid) and the targets are sent to the microbeam for irradiation. After irradiating the cells of the first FOV, the dish moves to the next FOV. The process is repeated until all cells in the selected dish area are irradiated.

As soon as the irradiation process finishes, the beam stops. The follow-up of irradiated cells is achieved through time-lapse bright-field imaging and cell tracking. Depending on the cell cycle duration, cells should be ideally followed for at least four cell cycles in order to detect division abnormalities in the progeny of the irradiated cells. Time-lapse imaging of the previously irradiated area is performed every 10 min.

#### Cell Detection in Bright-Field Microscopy

Although cell detection techniques have been described in the literature, these are dedicated to phase contrast (42, 44) or fluorescence imaging for microbeam irradiation. Phase-contrast image processing is based on the notion that cells are bright and the background is dark. Therefore, general image processing tools, such as thresholding, morphological processing and shape detection can synthesize a reliable pipeline through which cell detection is achieved (42). However, bright-field cell images suffer from certain drawbacks. They usually exhibit very low cell visibility and they include not only cells but also debris. Also, the use of polypropylene as substrate generates characteristic “loop” artifacts that severely interfere in both cell visibility and cell detection. Therefore, a special cell detection method was developed.

The cell detection method for microbeam targeting in bright-field imaging has been already analyzed (33) but a brief description is given in this paper. Images are acquired in a weakly defocusing mode (i.e., ±2–4 μm from the perfectly focused plane) in order to enhance cell visibility, which is a standard contrast-enhancement technique in bright-field illumination mode (45). MATLAB^®^ (The MathWorks, Natick, MA, USA) is used as software platform to design the cell detection module. Apparent cellular features originating either from the nucleus or the cytoplasm are detected using the Harris corner detector (46). This feature detection technique presents high selectivity in cellular features, while it limits substantially the detection of artifacts and background features.

The increased cell feature selectivity leads to using clustering techniques for grouping corners and forming cellular representations. Agglomerative hierarchical clustering is used to eliminate outlier corners, while a density-based technique groups the remaining corners capitalizing on their high density in cellular areas. Weighted centroids are calculated as *x–y* coordinates that are used as targets for irradiation or as cell markers for ensuring cell existence post-irradiation.

#### Cell Tracking and Division Detection in Bright-Field Microscopy

Cell tracking was achieved by using a detection-based technique called two-point microrheology (47). Cells are sequentially detected in all time-lapse images as described in Section “Cell Detection in Bright-Field Microscopy.” Each detected point *x–y* corresponds to one cell. Through cell tracking, each cell position is propagated in all time-frames by searching for its spatially nearest point in the following frames. Moreover, each cell is examined concurrently with its spatially nearest cell in order to avoid errors due to trajectory mixing during position linking. The linking depends on one input parameter that is the maximum predicted distance (in pixels) traveled by cells between two successive time-frames.

The cell tracking module has been adjusted in order to provide either off-line tracking after the completion of time-lapse imaging or on-line tracking in between successive time-lapse acquisitions. Using the latter, individual cell revisiting is possible in order to inspect the cell response to radiation in real time or even re-irradiate specific cells.

A critical requirement of this method is the ability to detect cell divisions in bright-field time-lapse images since these events determine the clonogenic potential. Detection of cell division is achieved through integrating a hybrid method. First, the number of cells is counted between two consecutive time-lapse acquisitions. The site in the dish where a candidate new cell appears is recorded as a possible site of division. Then, for each cell, the α-shape (48) or concave hull is calculated in order to provide a rough estimation of the cell outline. This calculation is based on connecting the outside corners that belong to a single cell. From the cell outline, the cellular area is calculated. Using the estimated cellular area from the α-shape and the number of corners attributed to this cell, a new parameter is defined as the corner density *d* per 100 pixels, for each cell, described by Eq. 5:

(5) $$d=\frac{\text{number of corners}}{\text{area in pixels}}\times 100.$$

**Figure 2:**
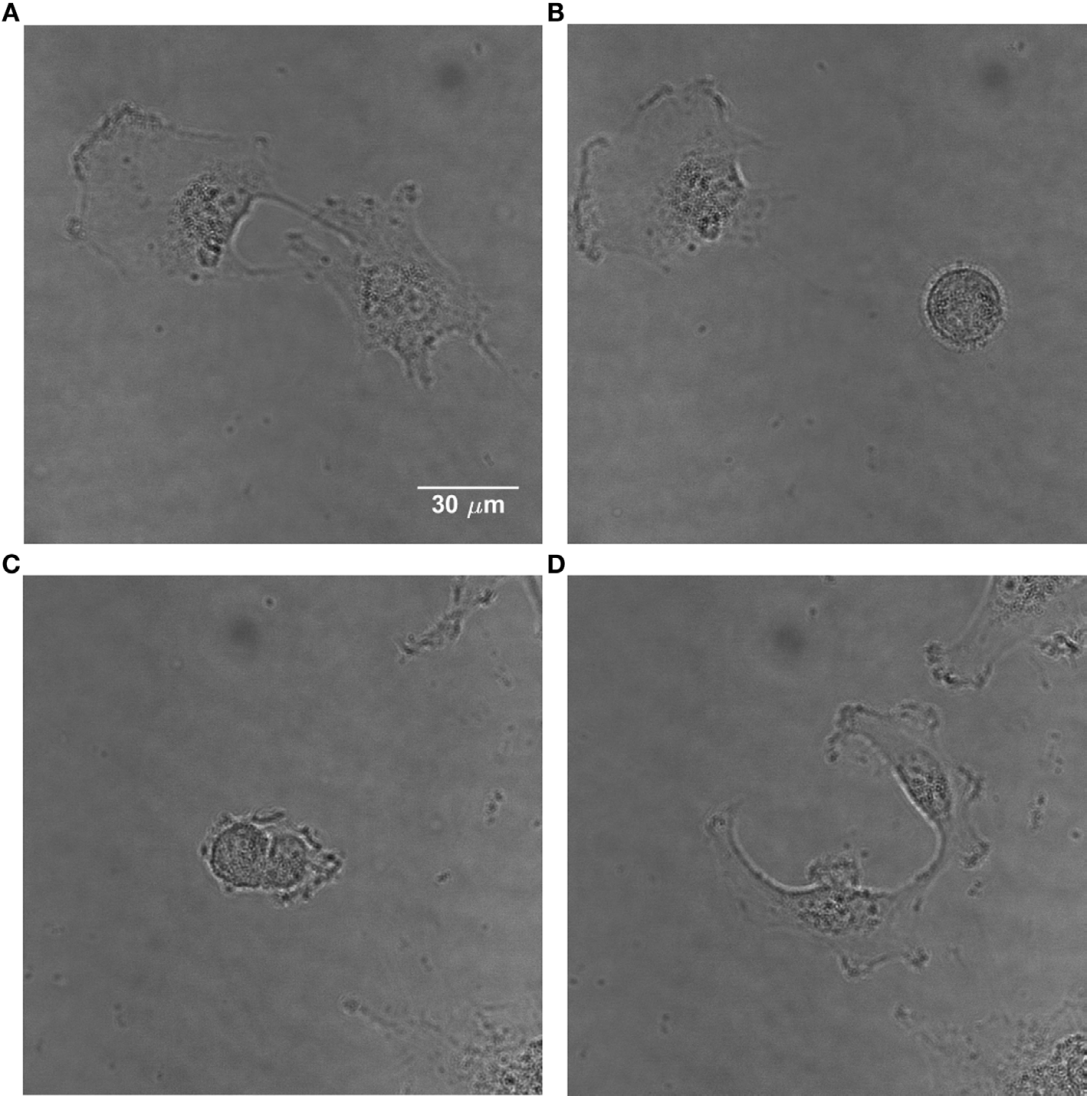
**Time-lapse images of U251 cells showing the change in morphology of a dividing cell**. **(A)** Non-dividing adherent U251 cells. **(B)** One of them divides, obtaining a characteristic round shape. **(C)** The dividing cell starts the separation process. **(D)** The cell has divided and the daughter cells become adherent.

Apart from the corner density, the eccentricity is calculated for each cell; this parameter describes the cell shape (49). It is well-known that mammalian cells obtain a characteristic elliptical or even round shape with highly condensed material when they intend to divide (Figure 2). Therefore, the eccentricity *e* is calculated according to Eq. 6, characterizing a cell as dividing or non-dividing:

(6) $$e=2\frac{\sqrt{{\left(\frac{M}{2}\right)}^{2}-{\left(\frac{m}{2}\right)}^{2}}}{M}$$

where *M* is the major and *m* is the minor axis of the potential ellipsis. A similar measurement of compactness has been also used by other researchers (42).

#### Evaluation of Clonogenic Potential

Following the cell detection prior and post-irradiation as well as the calculation of corner density and eccentricity, the next step is the calculation of the clonogenic parameters. The control dish is examined after 3–4 days, depending on the cell cycle, and the PE is calculated based on Eq. 7. Concerning the SF, this is calculated based on the number of cells that divided twice post-irradiation and not on the colony formation. Therefore, the SF is defined by Eq. 8:

(7) $$\text{PE}=\frac{\text{Number of cells that formed colonies}}{\text{Number of cells seeded}}$$

(8) $$\text{SF}=\frac{\text{Number of cells divided }N\text{ times after irradiation}}{\text{Number of cells seeded}\times \text{PE}}$$

In this case, the SF resembles another measurement, the mitotic index, which is the ratio of successfully divided cells to the total irradiated cells (50).

## Validation of Proposed Method

### Cell Detection

**Figure 3:**
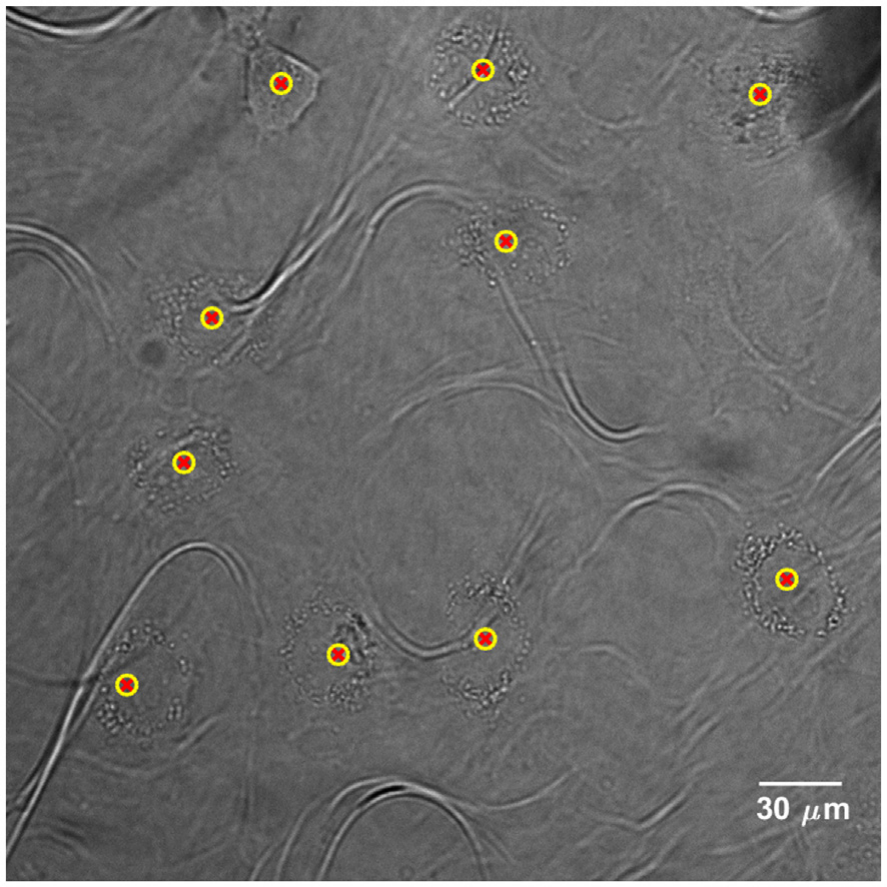
**Cell detection application on bright-field image of HeLa human cervix cells, acquired with a 40× objective**. Yellow-red markers define the *x*–*y* positions that characterize the cell presence. The “loop” artifacts originate from the polypropylene substrate since it becomes transparent in bright-field microscopy.

Cells are detected for each FOV and their positions are recorded in a list. The latter is updated every time a new cell is detected. Figure 3 shows the application of the cell detection module on an image of semi-adherent HeLa human cervix cells, obtained with a 40× objective. The density of cells in this area is higher than the optimum one.

### Cell Tracking and Cell Division Detection

**Figure 4:**
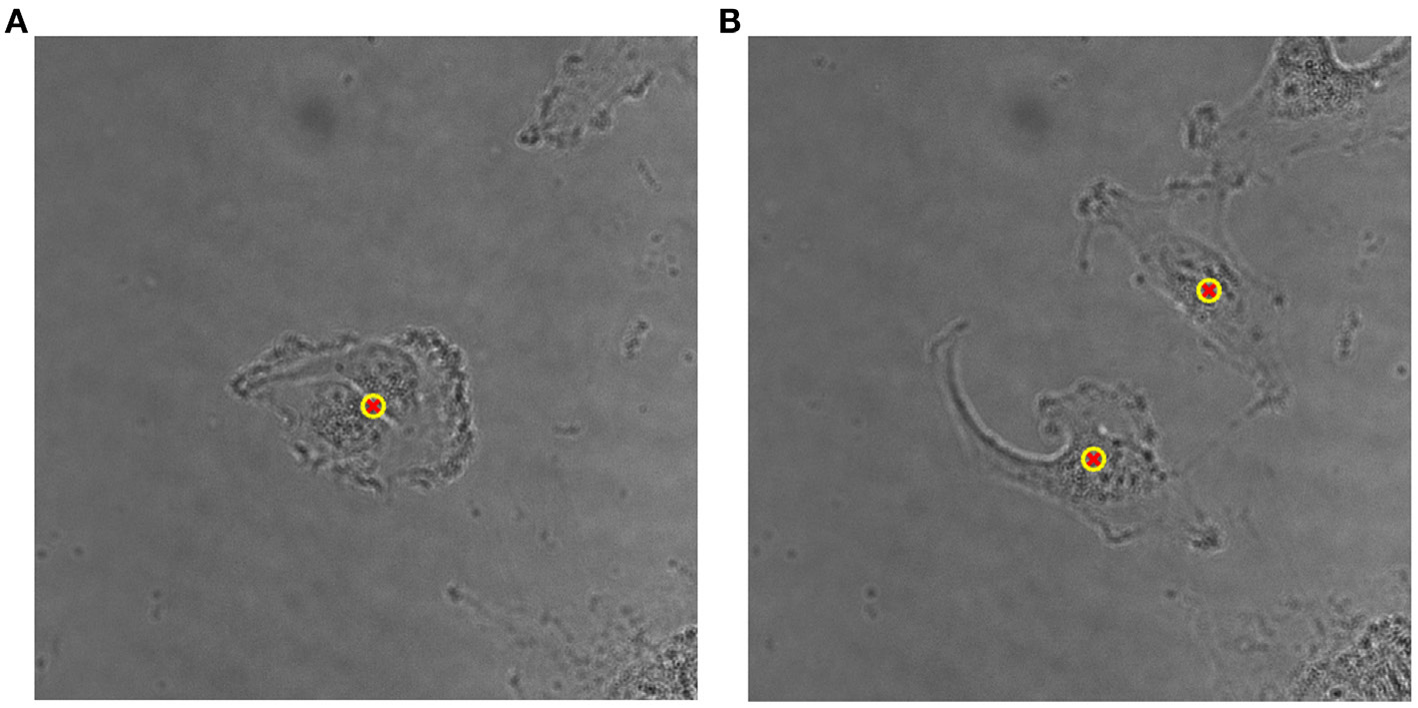
**(A)** Detailed view of original bright-field image of U251 cells on glass substrate, taken with 40× objective, with a single (parent) cell detected, indicated by an overlaid yellow–red marker. **(B)** Detection of two daughter cells post-division. The time difference between the two frames is 50 min.

**Figure 5:**
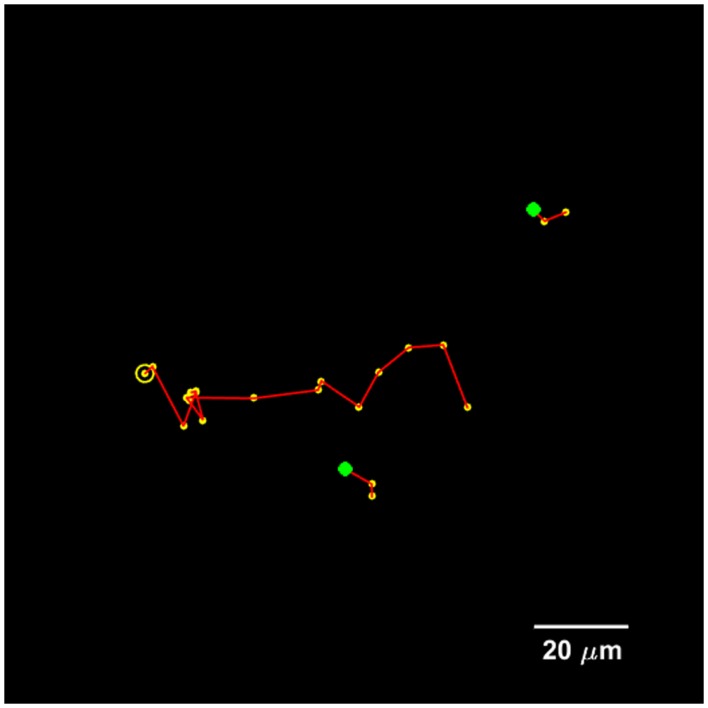
**Tracking diagram of the U251 cell(s) that are present in Figure 4 for a total duration of 20 frames, which corresponds to 3.5 h**. The yellow circle indicates the initial parent position, each yellow marker indicates subsequent positions, and the green markers indicate the initial positions of the daughter cells.

**Figure 6:**
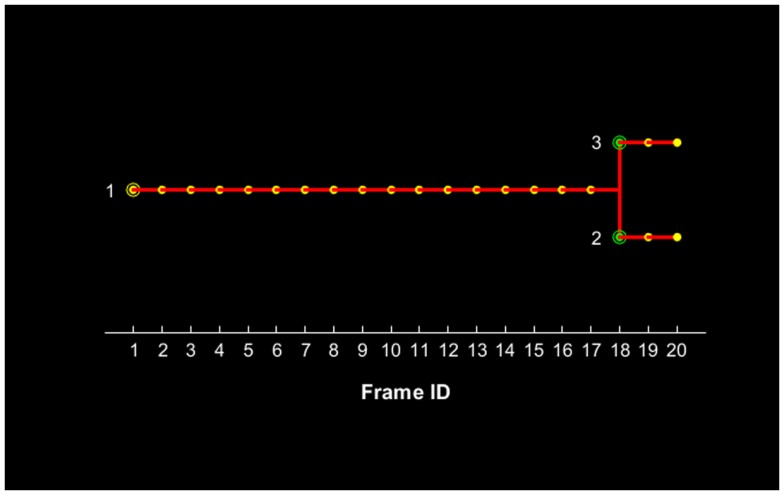
**Lineage tree of the U251 cell(s) presented in Figure 4 for a total duration of 20 frames, which corresponds to 3.5 h**. The initial parent cell (#1) position is denoted with a yellow circle and its presence is recorded for each subsequent position up to frame #17. After this frame, the parent cell is not recorded and the two daughter cells appear with their first record denoted with green circles.

The proposed method for cell tracking and cell division detection was tested on images of V79 Chinese hamster cells on polypropylene substrate. No errors were detected but the sets of images did not contain any divisions. Therefore, the module was tested on images of U251 cells on a glass-bottomed dish. Figure 4 shows the detection of two daughter cells (right, with red–yellow markers), originating from a single parent cell (left). Figure 5 shows the tracking diagram of the cell(s) of Figure 4, where the motion pattern can be identified while Figure 6 shows their lineage tree. The latter provides all the data needed to successfully identify a division: the two daughter cells are associated with a specific parent cell while the system records the time and frame at which the two cells were detected as separate entities.

**Figure 7:**
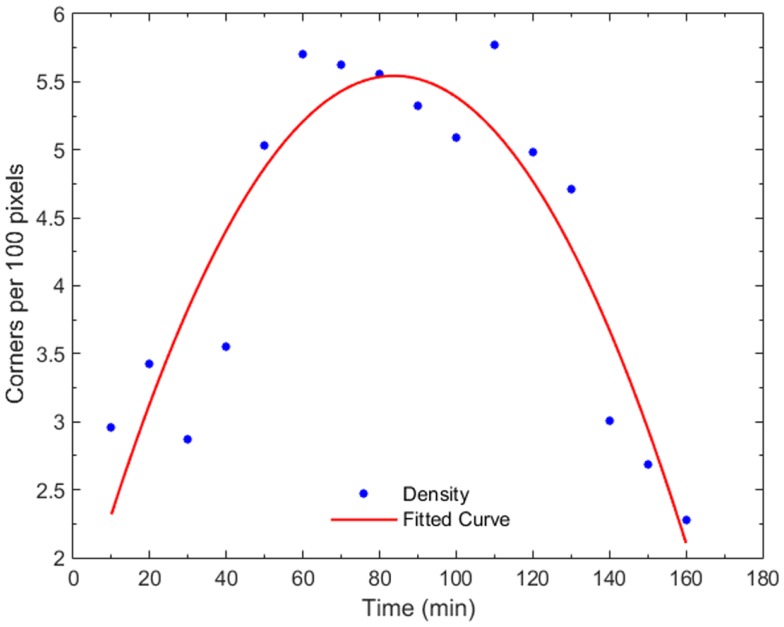
**Plot of corner density with time, for a dividing cell and one of its daughter cells**. In early mitosis, the corner density increases substantially (*t* = 50–80 min) since the cell becomes round with high-frequency internal details. At the cytokinesis stage, the density drops while, post-mitosis (*t* = 140 min), the density drops to non-dividing value because the cell expands.

Figure 7 shows the progression of corner density in the parent cell of Figure 4 and the corner density of one of the daughter cells. Corner density takes value in the range of 3.0–3.5 per 100 pixels for adherent cells while it reaches values higher than 5.5 in the actual cytokinesis process. At this stage, post-division, corner density decreases again for the daughter cells as soon as they become adherent again.

## Discussion

The use of bright-field illumination instead of the more commonly used fluorescence excitation prevents the induction of excess photo-toxicity and it avoids photo-bleaching effects. Therefore, the observation of cell reaction post-irradiation includes only radiation effects without effects originating from the toxic action of fluorescence stains. Although bright-field images are highly complex and cells become invisible in many cases, the cell detection method is successful at detecting at least 88% of the cells (33).

It has been well-understood from the early days of research with high-LET radiation that the latter generates linear survival curves with steep slope as a result of the high probability of cell killing, especially in the high-dose areas (14), while current evidence continuously confirms this notion (17, 19). However, very few cell types die soon after irradiation, through a programed death path. Research has shown that although mitotic index reached a minimum value at 4 h post-irradiation, cells may start to divide after this period of time (51). Most cells die when they attempt to divide since they cannot complete this process. Some cells may even divide successfully but they may bequeath hereditary effects that may cause death to the progeny. Therefore, it is essential to develop and/or integrate a cell tracking module that can track cells through time and detect divisions for more than one cell cycle. The assessment of mitotic catastrophe can enhance the knowledge of cell response to radiation and complement the colony formation assay.

The cell tracking module is effective on connecting cell trajectories. It is independent to the cell detection module since it connects only points and not entire cell structures. Therefore, it can be used to link trajectories for any cell detection method. The dependence of this module on only one input parameter makes the tracking application less complicated. The individual on-line cell tracking gives the opportunity for automated revisiting of cells at any time-point during the time-lapse imaging process in order to inspect or even re-irradiate one or more cells.

The cell division detection module bases its application on the cell appearance during the crucial division process. Cells obtain a more distinct appearance that makes their detection easier even in complex bright-field images. Their condensed material provides a highly textured view that produces a high number of more closely located corners than that of the adherent cells prior to cytokinesis. This texture gives a sharp increase in corner density, indicating a possible site of division. The division is confirmed by the sharp decrease of the eccentricity value: cell shape approximates an ellipsis or even circle and eccentricity approaches a value close to 0.

## Conclusion

A new method for clonogenic survival assay using high-LET microbeam radiation was proposed. The low probability of cell survival post-irradiation with high-LET particles shifted the clonogenic potential from colony formation to successful division of the progeny of irradiated cells and assessment of mitotic catastrophe. Cell tracking in bright-field illumination time-lapse images may provide a mechanism for high-throughput assessment of radiation response using stable cell-culture of patient-derived material.

## Author Contributions

All authors contributed to the revision and the approval, and agreed with this work. AG contributed to the conception and initiated this work, MM contributed to the conception of this work and performed image acquisitions, JJ performed cell dish preparation and imaging, NM performed cell dish preparation, NP and RB assisted in image acquisition while RJ had the overall overview of this work.

## Conflict of Interest Statement

The authors declare that the research was conducted in the absence of any commercial or financial relationships that could be construed as a potential conflict of interest.

## Abbreviations

FOV, field of view; LQ, Linear-Quadratic; LET, linear energy transfer; PE, plating efficiency; SF, survival fraction; U251, U-251 MG pleomorphic human glioblastoma (cells); WSVM, Wolfson Surrey vertical microbeam.
